# More Voyages in Vacation.—II

**Published:** 1890-02-01

**Authors:** 


### More Voyages in Vacation.—II.

Off Gibraltar, *January 2nd.*

This has been rather a bad day, wet and squally. The cloud effects have been very pretty, though, ending up with a fine sunset, just as we passed Cape Finisterre. I should like to have sketched it, with the squall for a black background, and the mist shrouding part of the mountains behind the cape, which loomed dark on the bright water, with its little, gleaming, white lighthouse on the top, while in the foreground was a vessel going our way—which, of course, we promptly raced, overtook, and left behind.

The boat is quieter now—but dinner has been varied by collisions of waiters, and headlong descents of plates piled on the sideboard—strange to say, they were not broken—and various other mishaps. The captain's sister is on board, but is too ill to appear at table. Her cabin is next mine, and I have paid her sundry visits. Some of the people are extremely ill, and the antipyrin does not appear to be so efficacious as it has been on former voyages. But I don't at all imagine that anyone is at the last gasp, as they seemed to be on one voyage, when somebody was overheard to say : " Oh, Tom, *do* forgive me before I die ! "

I have just been interrupted by a chat with the doctor, who tells me he has not as yet tried antipyrin after all. He has been using cocaine in preference—indeed, he has had to try it on himself, poor man. Nobody likes to admit themselves ill from sea-sickness, so he has been spending quite a quarter of an hour explaining to me that he simply suffering from an ordinary bilious attack.

I see by the log-book that the thermometer was 55deg. to-day, at twelve o'clock. I simply don't believe it! And when I indignantly accused Mr. Jaques, the second officer, of having made it warmer to please the people who complain of the cold, I am very much afraid he positively winked.

March 3rd.—If she won't be happy until she gets it, she ought to be perfectly happy now, for she *has* got it—lovely warm sunshine, tempered by a balmy breeze, and such an exquisitely blue sky and sea. The thermometer really is fifty-five to-day—in the shade, too—as Mr. Jaques took care to mention in the log-book, while in the sun it is sixty. It is almost unkind to mention this beautiful warmth when I know you are freezing in that March north-easter, of which we have a lively specimen coming down the Channel.

This morning we had rather a scratch service; the clergyman, feeling rather ill, cut it short. The captain, who had a sore throat, could not be present, while the lady who plays was forced to use the piano because the music-stool of the harmonium is not fixed and, in spite of the support of her husband on one side and mine on the other, she persisted in tumbling over at rehearsal, a proceeding, we judged would be unseemly at service. The singing was admirable; the doctor astonished us by coming out with a tremendous bass voice, which kept us in order and covered deficiencies.

To-morrow we are to be at Gibraltar, which is a day earlier than we were approximate time-tabled to be. This afternoon we have passed Lisbon, and had a magnificent view of Cintra—that exquisite castle in the air. It looks grander than ever from the sea-level, towering up in the most fantastic manner, castle and cliff so alike that one can hardly tell which is turret and which is natural pinnacle. The country looks barren and wild, not much verdure as yet, only a visible swelling of the twigs to be seen. The Victorians are all beginning to appear on deck, but I am "boss" among the ladies, being the oldest inhabitant of the yacht. To-day I had even to arrange the service, the captain not being there. His sister is better, and has been on deck, so has the young lady who has been hidden until now. Rumour said she was a beauty, and we all, with one accord, flocked to offer her deck chairs, and so on. Rumour was very wrong, however, and we were sorry we spoke! It was so rough last night that I was awakened several times by the swinging and banging of doors and the riotous behaviour of sundry waves that had gone and lost their way, and were careering over the deck. We have a descendant of Nelson's on board—a gilded youth of about seventeen, whom we have christened "The Admiral." He looks lonely, and has been very ill; Cape St. Vincent and Trafalgar awake no interest to him—in fact, he threatens to get off at Gibraltar and go home by rail—like the "four children who sail'd quite round the world, and came back by land on the other side." We "made our numbers" as we passed Lisbon Rock, and our having passed will be in all the papers to-morrow. We have had porpoises playing round, and plenty of gulls flapping overhead. They say there are flying fish in the Mediterranean, and that we shall meet the same swallows in Algiers of which we took leave in England last September.

The cooking and attendance are both excellent this time—there is a new chief-steward and two English cooks—everything is well done and tender, not messed up with butter and gravy in the sham French style. We get good dinners, hot and properly dressed, as well as properly served; what more can we want? Everyone seems satisfied, but, of course, that cheerful state of content will not last forty-two days. Just at present, other matters than "the hoith of good living" are occupying our thoughts, for Spain and Algiers are in sight, and we shall reach Gibraltar Straits in a few hours.

March 6th.—We had a most charming day at Gibraltar, yesterday. The sunset was magnificent as we were nearing the rock, and the mountains of Africa, which are covered with snow, looked perfectly sublime in the rosy light. Think of African mountains with snow on them! What is the good of the swallows going all that way to come to ice again? It went on getting warmer all day until we had to take off jackets, and put on shady hats, after which we simply basked in the sun. Let everyone who hankers after, or whose health requires, such glorious warmth follow our example by taking an ocean cure to the Mediterranean, and dodging the blighting bleakness of our celebrated English spring.

We arrived too late to go on shore that evening, so one of the ladies and I decided to do so by the nine o'clock boat next morning. She is very amiable, and her husband extremely amusing. He tells us the most entertaining stories. For instance, there is a whimsical old man on board who causes much exasperation among the others by always trying to steal their baths. He will not say what time he will have his, but waits until one has been turned on for somebody else, then slips in like a hermit crab, locking the door, if he have time to do so before the real owner comes. If not, there is trouble, and the *tableau* is delightful. Real owner: "Hi! you old vagabond, come out! That's my bath!" Old vagabond left standing outside, and found again twenty minutes after discoursing about baths to the desert air.

But to return to our day at Gibraltar. We went on shore at nine, and, having a pass to admit us, twelve gentlemen and two ladies, to the galleries, we had all to keep together. I was interested in the "works," but it was colder on shore, besides being wet and dirty. As a rule, the climate is excellent from November to May, but the rest of the year is hot, and the Levanters, or east winds, are disagreeable. The views from each embrasure were lovely, the blue Mediterranean far away, the flat ground of the Spanish and the British lines, the Neutral Ground, a low sandy isthmus, and the cemetery far below us, all blazing in the sunshine, and framed by the cold, dark wall of the cave, made a striking picture. But on the whole I am glad I have done those galleries; I shall never want to do them again. Then, we toiled up, and up and up a rugged pathway cut out of the solid rock. The signal-station is closed, at present, because new fortifications are being made which no one is allowed to see, in case they should report them to the enemy. And the hut on the opposite side of the hill is also in limbo, for the same reason, so that, practically, the only good views, of the rock were not on view.

However, we went on climbing, determined to go as high as we could and trust to Providence. We met several sentries who, examining our pass, let us proceed until we came to the entrance to the last path up to the signal-station where, as good luck would have it, we met a man who evidently could not read! Taking our paper, he scanned it—looking very wise—then, handing it back, he allowed us to go on—to our intense delight. So we reached the hut from which we had a grand view all over the Spanish mountains and away to Africa, as well as over the sheer precipice down to the blue, blue Mediterranean. From thence, we descended without so much as glancing at the concealed fortifications, and then up to the signal-station—our highest ambition, the point we were supposed not to be permitted to reach—but how we fared and what we saw must be reserved for our next.

